# An Unexpected GIST Causing Life-Threatening Bleeding after an Elective Hernia Repair

**DOI:** 10.1055/s-0042-1760130

**Published:** 2023-02-03

**Authors:** Athary Saleem, Fatemah Husain, Reem Boushehry, Mohammed Alshamali, Emad Fahim, Khaleel Mohammad

**Affiliations:** 1 Department of General Surgery, Al-Adan Hospital, State of Kuwait

**Keywords:** gastrointestinal stromal tumor, GIST, upper GI malignancy, wedge resection, inguinal hernia, case report

## Abstract

**Background** Gastrointestinal stromal tumors (GISTs) are the most common mesenchymal neoplasms of the alimentary tract. They are usually manifested by GI bleeding.

**Case Presentation** A 53-year-old male patient was admitted to the hospital for elective inguinal hernia repair. The patient did not have any history of GI symptoms in the past. A day after open inguinal hernia repair, the patient developed recurrent attacks of hematemesis resulting in hemodynamic instability and admission to the intensive care unit. An upper GI endoscopy identified a small bleeding gastric lesion. After multiple failed attempts to control the bleeding endoscopically, an emergency exploratory laparotomy was performed. An unexpected large fungating bleeding gastric mass was detected. The mass measured approximately 40 × 30 cm, and multiple peritoneal deposits were also discovered. A wedge resection of the anterior gastric wall along with the mass was performed. Histopathology revealed a high-grade (G2) GIST.

**Discussion** GISTs appear in variable sizes and may lead to a variety of complications including abdominal pain, GI obstruction, and bleeding. This case highlights the unexpected presentation and sudden bleeding of a large GIST in a totally asymptomatic patient undergoing elective hernia surgery. It also illustrates that GIST can be asymptomatic and grow to large sizes before developing clinical manifestations.

**Conclusion** The case report highlights a common complication of GIST with unexpected timing, immediately after routine hernia surgery.

Stromal tumors originating from the smooth muscle of the gastrointestinal (GI) tract such as leiomyoma, leiomyosarcoma, and bizarre leiomyoma have been historically described by Stout et al 1 2 . The name gastrointestinal stromal tumors (GISTs) later replaced those terms after the discovery of electron microscopy in the 1970s and immunohistochemistry in the 1980s. 1 2 GIST is a mesenchymal tumor that is derived from the interstitial cells of Cajal (ICC). It is associated with the overexpression of the *KIT* (KIT protooncogene receptor tyrosine kinase), *PDGFRA* (platelet-derived growth factor receptor alpha), and *SDH* (succinate dehydrogenase) genes. 1 3 4 Clinical manifestations vary based on their location, although one must keep in mind that 20% are asymptomatic. 1 3

GIST can metastasize to the liver and serosa or peritoneum, but rarely to the lymph nodes. 3 5 The initial diagnostic evaluation of GIST is based on imaging, including computed tomography (CT), magnetic resonance imaging (MRI), and endoscopic ultrasound (EUS). GI endoscopy also plays an important role in visualizing tumors within the foregut and colorectal regions. These tumors are usually submucosal, making it challenging to obtain an adequate biopsy for diagnosis through a standard endoscopy. Deeper biopsies are usually required, which makes EUS a valuable tool, especially when dealing with GISTs located in the foregut. In addition, immunohistochemistry and histopathology evaluation aids in GIST diagnosis. Morphologically, it is divided into three types: spindle cell, epithelioid cell, and mixed. Surgery and imatinib are the first-line therapy for GIST. 1 3 4 5

In this case report, we present an interesting clinical encounter of a patient presenting with life-threatening bleeding from a large gastric GIST after undergoing an elective open inguinal hernia repair. Our work has been reported in line with the SCARE 2020 criteria. 6

## Case Presentation

A 53-year-old male patient, with a medical history of hepatitis C virus and bilharzia, was admitted to the hospital for an elective inguinal hernia repair. He was asymptomatic on admission. Previously, the patient presented with right inguinal swelling and a right-sided testicular bulge that was noticed 3 weeks ago. He was evaluated by the general surgeon in the clinic. The documented abdominal physical examination by the clinic physician was incomplete and did not mention the presence of abdominal masses. Focused ultrasonography of the inguinal region was done and the patient was diagnosed with a right inguinal hernia. During the procedure, the intraoperative findings were: a huge varicocele, spermatocele, weak floor (weak transversalis fascia), multiple varicosities, and a lipoma of the cord. Unexpectedly, a hernial sac could not be identified. Excision of the right spermatocele and varicocele with tissue repair of the floor was performed. Postoperatively, the patient was doing well, with no specific complaints.

On the first postoperative day, the patient developed recurrent attacks of hematemesis resulting in hemodynamic instability and admission into the intensive care unit (ICU). Laboratory investigations were normal except for anemia ( Table 1 ). Next, an urgent upper GI endoscopy was performed. Due to the presence of large blood clots in the stomach, the gastroscopy could not be completed. Soon after that, a second trial revealed distal esophagitis with no varices and a deep bleeding ulcerated gastric mass measuring 3 to 4 cm in diameter located in the gastric body. The bleeding was controlled during the endoscopy by using endoscopic adrenaline injection and cauterization. Further imaging was planned to evaluate the bleeding source. Unfortunately, during the next 24 hours in the ICU, the patient developed another attack of hematemesis with hypotension requiring multiple blood products to be transfused.

**Table 1:** Laboratory investigations of the first hematemesis attack

Patient's vital signs	Laboratory investigations
GCS 15	CRP 94.8 mg/L
BP 90/50 mmHg	WBC 11.8 10 ^9^ /L
HR 115 beats per minute	Hemoglobin 92 g/L
RR 25 breaths per minute	Urea 5.2 mmol/L
SPO _2_ 98% in room air	Serum creatinine 67 µmol/L
Body temperature 37°C	Platelets 233 10 ^9^ /L
	PT 12.7 s

Abbreviations: BP, blood pressure; CRP, C-reactive protein; GCS, Glasgow Coma Scale; HR, heart rate; PT, prothrombin time; RR, respiration rate; SPO _2_ , saturation of peripheral oxygen; WBC, white blood cell.

**Fig. 1(a, b):**
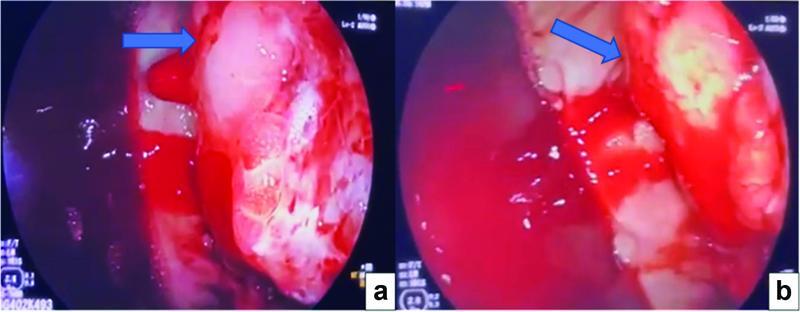
A limited endoscopic view (preoperative) of an intraluminal mass with active bleeding as indicated by the *blue arrows* pointing to the mass.

**Fig. 2 (a–c):**
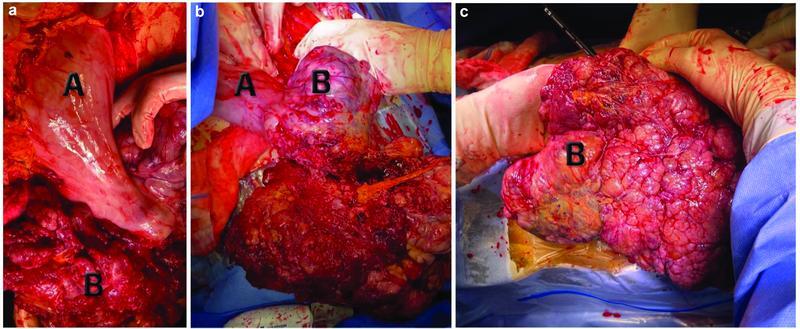
Surgical exploration revealing a pedunculated fungating mass arising from the gastric fundus and body at the anterior surface. A: stomach; B: gastric mass (GIST).

**Fig. 3:**
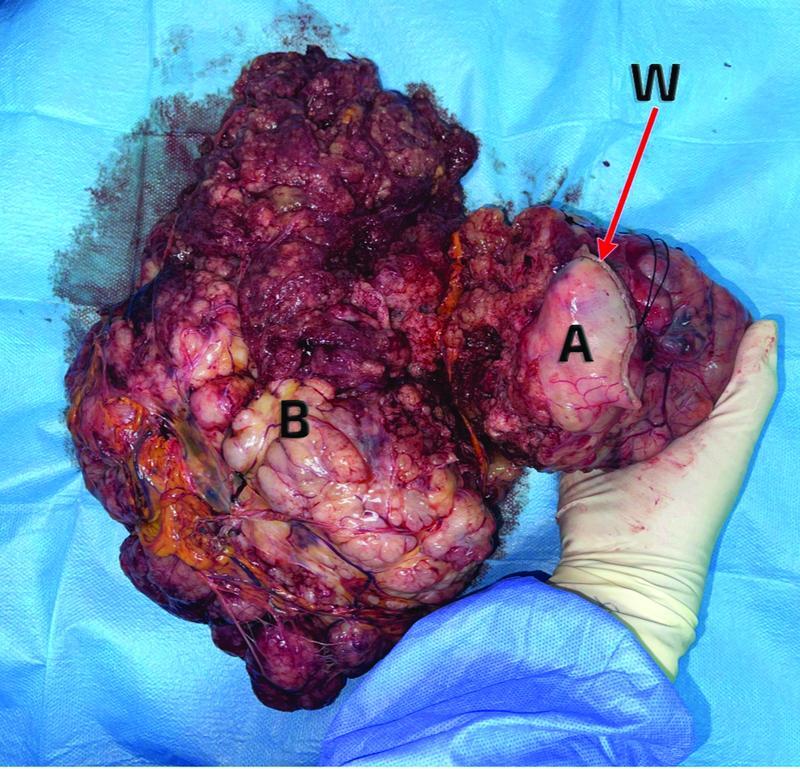
The resected mass, where A is the stomach, B is the fungating mass, and W is the gastric staple line from the wedge resection.

**Fig. 4(a–c):**
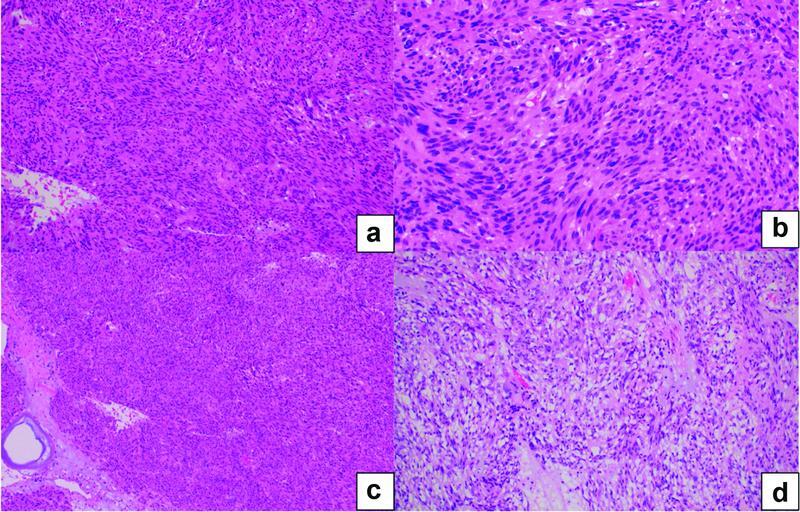
Spindle cell tumor with blind-looking nuclei. There is no mild atypia with very rare mitotic figures. **(d)** An epithelioid component.

**Fig. 5:**
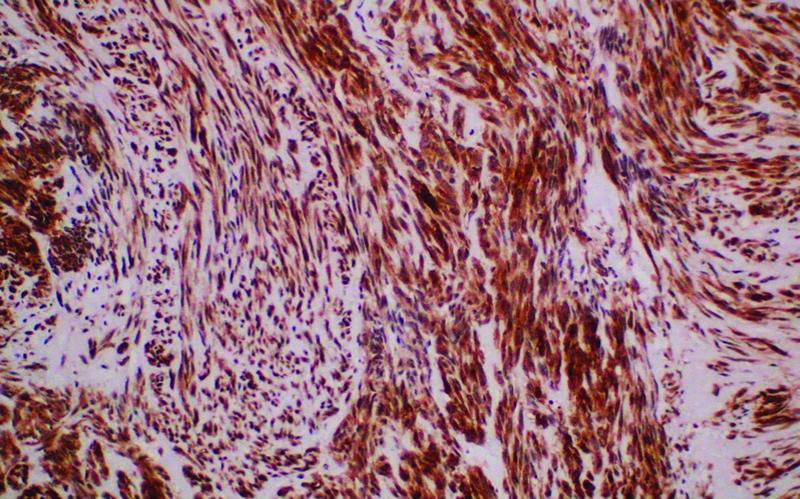
CD117 positive.

Endoscopy was attempted for the third time, which was unsuccessful at controlling the bleeding source ( Fig. 1 ). The patient was then taken to the operating room for an emergency exploratory laparotomy. A large pedunculated bleeding fungating gastric mass, originating from the anterior gastric wall, was found, measuring approximately 40 × 30 cm ( Figs. 2 and 3 ). Significant hemoperitoneum was also observed along with multiple peritoneal deposits. A wedge resection of the anterior gastric wall along with the mass ( Fig. 3 ) was performed, leaving behind healthy gastric margins. Intraperitoneal deposits were detected on the upper rectum, bowel, and anterior abdominal wall and were sampled and sent for a histopathology examination. The patient had a progressive and uneventful postoperative recovery in the ICU and the ward and was discharged home in 1 week. Histopathology revealed a high-grade (G2) GIST with spindle cell type and peritoneal deposits. The spindle cells were positive for CD117 and negative for pan-CK (pancytokeratin immunohistochemistry biobsy tissue for pancytokeratin), S100, desmin, and SMA (smooth muscle actin) ( Fig. 4 and Fig. 5 ).

## Discussion

GIST is a rare tumor accounting only for 0.1 to 3% of all GI malignancies. 1 3 6 The pathogenesis of this tumor occurs within its myogenic and neurogenic components as a result of *c-KIT* dysfunction and ICC hyperplasia, respectively. 3 4 6 More than 90% of GIST demonstrates overexpression of the receptor tyrosine kinase KIT (CD117). 3 4 7 Morphologically, GIST has three types. These include spindle cell type (70%), epithelioid type (20%), and mixed type (10%). The spindle cell type usually arises from the stomach or small intestine and appears as a huge heterogeneous mass, enhancing radiological imaging. 3 6

The majority of GISTs are located in the stomach, followed by the small bowel, and are least common in the colorectum and esophagus. In addition, it may also arise from the mesentery, omentum, and peritoneum. Extragastrointestinal stromal tumors can also occur but are rare. 5 Common areas of metastasis include the liver, peritoneum, and omentum but rarely the pulmonary and abdominal lymph nodes. 3 Clinical manifestations depend on the primary tumor location and size. 3 4 6 8 The most frequent clinical feature is GI bleeding (GIB), which can be seen in about 30% of symptomatic patients 9 and may be associated with melena and/or hematemesis. It may also present with nonspecific signs and symptoms such as early satiety, anorexia, and abdominal distension. 3 4 5 6 10 While small GISTs (<2 cm) are usually asymptomatic and often detected incidentally, large tumors are symptomatic and can ulcerate, leading to GIB. 8

In spite of the large tumor present in our patient, he presented for an elective inguinal hernia repair with no GI symptoms. Postoperatively, he developed massive life-threatening hematemesis, which was the first significant presentation of the GIST. Even though bleeding is the most frequent clinical feature of GIST, significant acute bleeding is considered uncommon. 9 11 Because of GIST's high vascularity, large tumors expand their vascular supply and invade the mucosal layer of the nutrient vessels. This causes considerable areas of necrosis, ischemia, hemorrhage, and ulceration, resulting in intraluminal hemorrhage. 11 12 Therefore, intraluminal hemorrhage is the usual presentation in GISTs. 8 9 However, we would like to highlight that intraperitoneal hemorrhage is uncommon and can result from spontaneous tumor rupture and/or consecutive surgical trauma. 6 12 13 14 In our patient's case, the GIB was intraluminal as visualized endoscopically immediately prior to surgery, as well as intraperitoneal as identified during laparotomy, adding to its novelty.

Multiple studies highlight the importance, safety, and feasibility of the laparoscopic approach in managing GISTs in stable and elective cases. Laparoscopic surgery has gradually replaced open surgery in elective cases due to its advantages of less intraoperative blood loss and faster postoperative recovery. 15 Complete surgical resection with negative margins is the only curative treatment option for GIST. However, in the case of an unresectable or borderline resectable tumor as well as metastatic lesions, tyrosine kinase receptor inhibitors are indicated. In ideal situations, radiological investigations would have played an important role not only in preoperative diagnosis and staging but also in deciding neoadjuvant treatment. Due to the timing sequence and critical presentation of this patient's GIB, this was not possible. Nonetheless, this does not preclude the patient from the benefits of adjuvant chemotherapy given the findings of metastasis intraoperatively. The patient was transferred to a cancer care center where he started his chemotherapy journey with imatinib mesylate 400 mg tablet once daily. Following the surveillance protocol recommendations, 1 5 the patient was advised for clinical examination and an abdominopelvic CT scan every 3 to 6 months.

## Conclusions

In this reported case, the patient was initially asymptomatic, which is unusual considering the size of the GIST. Bleeding manifestations of GIST occurred coincidentally immediately after undergoing an elective inguinal hernia repair. The GIB was found to be both intraluminal and intraperitoneal, which is quite uncommon to occur simultaneously in a GIST. Emergent laparotomy revealed metastatic GIST, which warranted excision of the primary gastric tumor and subsequent further management at the cancer care center. Pitfalls in this case that serve as important learning points include the importance of a complete and thorough physical examination including a detailed and complete abdominal ultrasound as part of a routine hernia assessment to exclude other GI abnormalities, which can be easily missed. Furthermore, GIST may present in the immediate postoperative phase in the form of life-threatening GIB, which is important to consider when dealing with similar clinical encounters.
